# Exploring the role of fear of missing out in coping and risk-taking among alcohol use disorder and general young adult populations

**DOI:** 10.1016/j.abrep.2024.100532

**Published:** 2024-01-23

**Authors:** Diana Jaworska, Katarzyna Iwanicka

**Affiliations:** a Faculty of Psychology, University of Warsaw, Stawki 5/7, 00-183 Warsaw, Poland; b Faculty of Journalism, Information and Book Studies, University of Warsaw, Bendarska 2/4, 00-310 Warsaw, Poland

**Keywords:** FOMO, Fear of missing out, Alcohol use disorder, Coping, Risk-taking

## Abstract

A high level of fear of missing out (FOMO) is related to many adverse emotions and behaviors, including stress, maladaptive coping, risk-taking, and alcohol consumption. However, previous research on FOMO has not considered particularly vulnerable cohorts like individuals with alcohol use disorder (AUD). In this project, we have conducted two preliminary studies (N1 = 356; N2 = 66) investigating the role of FOMO in alcohol-related coping strategy in a general population of young adults as well as a clinical sample of AUD patients. Results showed that FOMO is a positive predictor of alcohol coping in both studied samples, and it is a positive predictor of health risk-taking among people with AUD. The study identifies high FOMO as a risk factor for adopting maladaptive coping strategies, specifically alcohol coping.

## Highlights

• FOMO is a positive predictor of alcohol coping in general young adult sample.
• FOMO is a positive predictor of alcohol coping in a sample of AUD patients.
• FOMO is a positive predictor of health risk-taking in a sample of AUD patients.

## 1 Introduction

Fear of Missing Out (FOMO) can be defined as the “pervasive apprehension that others might be having rewarding experiences from which one is absent” (Przybylski et al., 2013). This phenomenon is widespread among the younger population and leads to significant negative consequences across different aspects of life (Elhai et al., 2021, Jupowicz-Ginalska et al., 2021). Individuals characterized by pronounced FOMO encounter a plethora of adverse feelings, such as depressive symptoms and anxiety, and may adopt diverse strategies to manage these sentiments (Dempsey et al., 2019, Elhai et al., 2020). This research posits that those with elevated FOMO frequently resort to maladaptive stress-coping mechanisms, with a specific emphasis on an increased propensity to alcohol coping. This study directs its attention towards both the broader demographic of young adults and a clinical cohort undergoing treatment for alcohol use disorder (AUD).

FOMO has been examined from various theoretical angles (see for a review Çelik et al., 2022). Among the prominent theories employed to elucidate FOMO is the Self-Determination Theory (SDT; Deci and Ryan,1985). Simultaneously, this theory could be the most applicable for explaining the role of FOMO in maladaptive stress-coping mechanisms (alcohol consumption in particular). SDT suggests that to effectively self-regulate and maintain mental well-being, it is essential to fulfill three fundamental psychological needs: competence, autonomy, and relatedness. As noted by Przybylski et al., (2013), individuals with consistently low levels of satisfaction in these basic needs may become more attuned to the fear of missing out. Consequently, they may turn to social media as a means of compensating for these unmet needs. Given that an elevated FOMO is linked to potential misuse of social media and correlates with various undesirable traits and behaviors (e.g., Blackwell et al., 2017, Elhai et al., 2020, Sekścińska and Jaworska, 2022), it can result in significant repercussions across various aspects of life. When looking at the FOMO explanation in SDT framework, it is plausible that the use of social media might be just one among several self-regulatory behaviors that individuals with high FOMO engage in. We posit that another self-regulatory behavior of people with high FOMO might be restoring to alcohol consumption to cope with negative emotions.

Coping restores to use of cognitive and behavioral strategies to manage stressful situations (APA, 2018). One commonly observed maladaptive coping mechanism is using alcohol to deal with negative emotions, a behavior referred to as alcohol-related coping (Carver et al., 1989). It appears essential to particularly focus on coping as a driving force behind drinking, as it has been linked to more detrimental outcomes associated with alcohol consumption (for a review see Kuntsche et al., 2005). Relying on alcohol to cope with negative emotions is closely linked to alcohol-related problems (McNally et al., 2003). The argument posits that although drinking for coping purposes may provide short-term relief, it leads to adverse long-term consequences as alcohol coping fails to address the underlying issues that contributed to negative emotions in the first place (Kassel et al., 2000). Given the potential severity of consequences for individuals, our studies specifically explore the role of FOMO in a tendency to use alcohol coping.

There is a positive relationship between FOMO and stress (Adams et al., 2020, Adrian and Sahrani, 2021). Research has shown that people with high FOMO less frequently use active coping strategies such as behavioral activity and engagement to deal with negative emotions (Elhai et al., 2016). Moreover, FOMO is associated with increased alcohol consumption per session, suffering more severe consequences because of its overuse, such as engaging in dangerous risks or feeling embarrassed (Riordan et al., 2015). This link seems particularly strong as it holds even after controlling for personality factors (Riordan et al., 2023). FOMO was also shown to be related to greater typical weekly alcohol consumption and a lower age of onset (McKee et al., 2022) as well as to binge-drinking tendency among adolescents (Brunborg et al., 2022). Hence, the previous studies demonstrated a connection between FOMO and drinking. Nevertheless, the aspect of FOMO's role specifically in resorting to alcohol as a coping mechanism for stress has not been explored so far.

Moreover, it should be noted that most studies measuring the relationship between alcohol consumption and FOMO focused on the group of college students (Riordan et al., 2015, Riordan et al., 2021, Wolkowicz, 2016), not considering clinical groups. This may obscure the picture of this phenomenon, because, as research shows, students reach for alcohol often due to different motives than other adults, such as peer pressure or social and conformity motives (Iwamoto and Smiler, 2013, LaBrie et al., 2007). Motives for alcohol consumption might differ depending on many factors, e.g., gender, where sensation seeking is observed particularly among young men, while among women, the motivation for drinking to cope with challenging emotional states appears to play a more substantial role (Kuntsche et al., 2006). The motivational model suggests that drinking motives can stem from internal or external sources, and they vary in terms of the expected effects' valence. In other words, young people may drink to enhance positive feelings or alleviate negative ones (Kuntsche et al., 2005). However, the issue of whether FOMO is a significant predictor of alcohol-related coping in older adults remains unknown. As FOMO can increase the negative consequences of alcohol consumption, it is crucial to investigate its role also among older people. We believe that the clinical sample of AUD individuals necessitates particular focus as, coming back to SDT framework, this group might especially suffer from unmet basic needs (Lee & Kang, 2019). To the best of our knowledge, no studies have investigated FOMO in clinical samples of people with AUD.

Individuals with AUD are a particularly vulnerable population due to their low distress tolerance, which is why FOMO may have an especially strong effect on them (Zaorska et al., 2023). Research shows that AUD patients use non-adaptive coping strategies, such as those focused on emotions or avoidance, significantly more often than the general population (Ribadier & Varescon, 2019). In our research, we would like to investigate whether and to what extent FOMO can explain, why individuals with AUD reach for these maladaptive strategies, in particular alcohol-related coping strategy (alcohol coping). Studies have shown that e.g., abstinence period and duration of treatment improve coping strategies throughout the therapy (Cavicchioli et al., 2019). We investigate the role of FOMO while controlling also for abstinence period in a clinical sample.

People experiencing unsatisfied psychological needs, which is a key feature of FOMO, may try to look for some alternative ways to fill these emotional demands, sometimes even hazardous ones (not only covering drinking habits, but the health domain in general). Hence, this makes them more prone to get involved in risky behaviors. FOMO has a direct positive significant impact on risk-taking travel behaviors (Mohanan, & Shekhar, 2021), distracted driving (Przybylski et al., 2013), traditional gambling and stock market trading domains (Song, 2022) or academic misconduct (McKee et al., 2022). Also, people struggling with addictions are more prone to various forms of risk-taking (Dahne et al., 2013). However, until now, the role of FOMO in taking health risks in people with AUD has remained unknown. Studies have shown that college students high in FOMO engage in more health-risks, which might have negative repercussions for them (Riordan et al., 2015). Taking this into account, the role of FOMO in health risk taking among a clinical population should be examined.

## 2 The current study

In the current study, we aimed to investigate the role of FOMO in alcohol-related coping strategy and health risk-taking among a clinical sample of AUD patients, and a general cohort of young adults. Previous studies have already shown the significant role of FOMO in alcohol consumption among young adults (e.g., Riordan, et al., 2021). Yet so far, the research has highlighted the social and/or conforming motives for drinking alcohol among young adults high in FOMO. In the following project, we posit that alcohol coping might be a self-regulatory behavior of people with high FOMO levels, as the result of their unmet basic needs (following the framework of SDT). We incorporate a sample of clinical AUD patients alongside a sample of the general young adults to investigate whether the significance of FOMO in alcohol coping holds particular importance for a vulnerable population. Since FOMO has shown adverse effects on young adults, it is essential to explore this aspect among individuals with AUD, as they have fewer coping resources to deal with the emotional distress (Zaorska et al., 2023). Additionally, by including both populations in a single article, we can discuss a comparison regarding the role of FOMO in both samples, particularly by evaluating standardized regression coefficients. So far there have been no studies that would investigate the level of FOMO in this particular clinical group, nor the role of FOMO in explaining the maladaptive coping of people with AUD.

Based on the existing literature, we expected that:

H1. FOMO is a positive predictor of alcohol-related coping strategy in a general sample.

H2. FOMO is a positive predictor of alcohol-related coping strategy in a clinical sample.

H3. FOMO is a positive predictor of health risk-taking in a clinical sample.

### 2.1 Methods

#### 2.1.1 Participants and procedure

356 young adults from a general population (175 females; age: 18–36; *M* = 26.9; *SD* = 4.9) and 72 clinical patients with AUD (53 females; age: 21–65; *M* = 40.83; *SD* = 11.23) took part in our studies. The demographic characteristics of the sample are presented in Table 1. Study 1 on the general population was conducted on a research participant panel as a part of a larger survey. The remaining part of the survey was not related to the subject of this study, and it did not affect the results (participants filled in FOMO scale and Mini-COPE questionnaire in the first part of the survey). Participants were rewarded points that could be exchanged for prizes from the panel store. Clinical patients for Study 2 were recruited through posts on private social media support groups for people in AUD treatment. The inclusion criterion was a minimum of one month of abstinence due to severe symptoms of alcohol withdrawal that AUD patients often experience in that time, which might impede their reasonable judgment (McKeon et al., 2008) (the duration of abstinence in months in our sample: *M* = 40.14 *SD* = 70.45). As six people did not fulfill this requirement, we excluded them from the analyses, thus the final sample consisted of 66 people. This group did not receive any reward for their participation.

**Table 1:** Demographic sample characteristics.

x	Study 1				Study 2			
	*n*	*%*	*M*	*SD*	*n*	*%*	*M*	*SD*
Gender								
Women	175	49.2			53	73.6		
Men	181	50.8			18	25		
Other	0	0			1	1.4		
Education								
Primary	20	5.6			1	1.4		
Secondary	189	53.1			28	38.9		
Higher	147	41.3			43	59.7		
Place of residence								
Village	90	25.3			6	8.3		
City up to 50.000 residents	74	20.8			13	18.1		
City from 50.000 to 150.000 residents	58	16.3			10	13.9		
City from 150.000 to 500.000 residents	69	19.4			6	8.3		
City with more than 500.000 residents	65	18.3			37	51.4		
Age			26.9	4.9			40.83	11.23

Both studies were approved by the Ethics Board of Faculty of Psychology, University of Warsaw.

#### 2.1.2 Measures

***FOMO.*** The trait level of FOMO was measured with the use of the 10-item Fear of Missing Out Scale by Przybylski et al. (2013). Participants were asked to indicate how true each statement was to them on a scale from 1 (not at all true of me) to 5 (extremely true of me). The indicator of the FOMO level was the mean of the answers (the higher mean indicated a higher FOMO level). The reliability of FOMO scale was satisfactory in both samples (general sample: Cronbach’s alpha = 0.88; clinical sample: Cronbach’s alpha = 0.84).

**Alcohol-related coping strategy.** To measure alcohol coping, we used a Polish version of the Mini-COPE questionnaire (Juczyński, Ogińska-Bulik, 2009). It consists of 28 items, which cover a range of coping styles, including problem-solving, positive reframing, seeking social support, seeking emotional support, acceptance, humor, turning to religion, self-distraction, self-blame, behavioral disengagement, venting, substance use, and denial. In our study, we focused on the alcohol coping subscale, which has 2 items (I drink alcohol or use other substances to feel better.; I drink alcohol or use other substances, which help me get through that.). Participants are asked to rate how often they use each coping strategy on a scale ranging from 0 (almost never) to 3 (almost always). It is important to stress that, although the questions display the use of other substances as well, these substances are not named, therefore the main focus is on alcohol. For this reason, we refer to these answers as indicating the inclination to use alcohol-related coping strategy. The scores of each subscale are averaged to create one indicator of one’s inclination to use a given strategy. The reliability of alcohol coping measures (2-item subscale of Mini-COPE) was satisfactory in both samples (general sample: Cronbach’s alpha = 0.88; clinical sample: Cronbach’s alpha = 0.96).

**Propensity to health risk-taking** was measured only in Study 2 with one subscale of the DOSPERT Scale (Blais, Weber, 2006). The DOSPERT Scale consists of 30 statements relating to five domains of risk. The health subscale consists of six items (e.g., Consuming excessive amounts of alcohol in a social situation). Respondents are asked to indicate the likelihood that they would engage in the described behavior on a scale from 1 (very unlikely) to 7 (very likely). The indicator of participants’ propensity to health risk-taking was a mean of their scores. The reliability of DOSPERT Scale was satisfactory (clinical sample: Cronbach’s alpha = 0.73).

**Sociodemographic questions.** We controlled for participants’ age, gender, education, and place of residence, as well as alcohol abstinence period (only in the clinical sample).

The questionnaire used in the study can be found in Supplementary Material.

## 3 Results

### 3.1 Analysis strategy

The first step of analysis was conducting basic descriptive and correlation tests in both studied samples. Next, the linear regression model for the general sample was created, with alcohol coping as DV, and age, gender, and FOMO as IVs. Following that, two linear regression models were calculated for the clinical sample with the same IVs: age, gender, FOMO, and abstinence period. The first model included alcohol coping as a DV, and a second model included health risk-taking as a DV.

### 3.2 Correlations between the variables in both samples

The descriptive statistics for the variables can be found in Supplementary Material. Results of Person's r correlation analysis have indicated significant positive correlations between FOMO and alcohol coping in both studied groups (Study 1 general sample: *r* = 0.43, *p* <.001; Study 2 clinical sample: *r* = 0.35, *p* =.004). Moreover, FOMO positively correlates with health risk-taking in Study 2 (*r* = 0.36, *p* =.003). All correlations are presented in Table 2.

**Table 2:** Correlations between FOMO and coping strategies and health risk-taking in both studies.

		FOMO (Study 1)	FOMO (Study 2)
Alcohol coping	*r*	0.429	0.353
	*p*	<001	0.004
Self-blame	*r*	0.305	0.437
	*p*	<001	<001
Behavioral disengagement	*r*	0.437	0.338
	*p*	<001	0.006
Self-distraction	*r*	0.214	0.171
	*p*	<001	0.169
Denial	*r*	0.472	0.132
	*p*	<001	0.292
Emotional support	*r*	0.162	0.049
	*p*	0.002	0.697
Instrumental support	*r*	0.245	0.096
	*p*	<001	0.443
Active coping	*r*	−0.076	−0.083
	*p*	0.151	0.510
Planning	*r*	−0.018	−0.195
	*p*	0.735	0.116
Acceptance	*r*	−0.030	−0.239
	*p*	0.575	0.054
Positive reframing	*r*	0.123	−0.048
	*p*	0.020	0.699
Religion	*r*	0.293	−0.195
	*p*	<001	0.117
Venting	*r*	0.284	0.271
	*p*	<001	0.028
Humor	*r*	0.329	0.010
	*p*	<001	0.935
Health risk-taking	*r*	–	0.363
	*p*	–	0.003
N		356	66

### 3.3 The role of FOMO in alcohol coping in a general sample

To examine the role of FOMO and demographic variables in alcohol coping a linear regression analysis was conducted with age, gender (dummy coding, female = 1), FOMO as IV, and alcohol coping as DV. The proposed model is significant *(F* (3, 352) = 31.19; *R^2^* = 0.21, *p* <.001). Results have shown that FOMO is a positive predictor of alcohol-related coping strategy *(β = 0.42; p* <.001), which confirms H1. The detailed results are presented in Table 2.

### 3.4 The role of FOMO in in alcohol coping in a clinical sample

To examine the role of FOMO and demographic variables in alcohol coping in a clinical sample, we have conducted a linear regression analysis with age, gender (dummy coding, female = 1), FOMO, and abstinence period as IV, and alcohol-related coping strategy as DV. The proposed model is significant *(F* (4, 60 = 7.24; *R^2^* = 0.33*; p* <.001). Results have shown that FOMO is a positive predictor of alcohol coping *(β = 0.27; p* =.021), which confirms H2.

### 3.5 The role of FOMO in in health risk-taking in a clinical sample

Next, to examine the role of FOMO and demographic variables in a tendency towards risk-taking in the domain of health and safety, another linear regression was conducted with age, gender (dummy coding, female = 1), FOMO, and abstinence period as IV, and health risk as DV. The proposed model is significant *(F* (4, 60) = 3.34; *R^2^* = 0.18, *p* =.015). Results have shown that FOMO is a positive predictor of health risk-taking *(β = 0.42; p* <.001), which confirms H3. The detailed results are presented in Table 3.

**Table 3:** Regression analyses for both studies and both DVs.

Predictors	*B*	*SE*	*Beta*	*t*	*R2*	*Adjusted R^2^*	*F change*
*DV: Alcohol-related coping strategy (Study 1 – general population)*					0.210	0.203	31.198***
FOMO	0.092	0.010	0.421	8.829***			
Age	0.010	0.017	0.029	0.598			
Gender	−0.529	0.162	−0.156	−3.266**			
*DV: Alcohol-related coping strategy (Study 2 – clinical sample)*					0.325	0.281	7.238***
FOMO	0.427	0.180	0.269	2.378*			
Abstinence period	−0.006	0.001	−0.394	−3.335***			
Age	0.001	0.013	0.006	0.047			
Gender	0.439	0.286	0.170	1.534			
*DV: Health risk-taking (Study 2 – clinical sample)*					0.182	0.128	3.342**
FOMO	0.810	0.242	0.417	3.345***			
Abstinence period	−0.002	0.002	−0.100	−0.769			
Age	0.006	0.017	0.044	0.323			
Gender	−0.643	0.386	−0.204	−1.666			

*p <.05; ** *p* <.01; *** *p* <.001.

## 4 Discussion

Our primary objective in this project was to examine, how FOMO contributes to alcohol-related coping strategy and the propensity for health risk-taking behaviors. This investigation encompassed both a clinical group consisting of individuals with AUD and a broader population of young adults. The results have confirmed our hypotheses, revealing a positive predictive role of FOMO in alcohol-related coping strategy among both examined groups. Additionally, FOMO was identified as a positive predictor of engaging in health risk-taking behaviors among individuals with AUD.

Our findings indicate the link between FOMO and alcohol-related behavior (alcohol coping in particular), which is in line with previous studies showing the link between FOMO and alcohol consumption (e.g., Riordan et al., 2015). The originality of our study lies in the fact that it included the population of people with AUD. Our results also confirm the reports of Wolkowicz et al. (2023), which showed that FOMO was associated with the patients' experience of alcohol craving. It can therefore be assumed that, following Self-Determination Theory, FOMO is related to the unsatisfied basic needs, fulfillment of which is crucial for an individual's well-being. The frustration of this need, in turn, causes tension, which might turn into alcohol craving among AUD patients. Brunborg et al (2022) obtained results showing that adolescents with higher FOMO have greater risk of binge-drinking, explaining this relationship, by the fact that FOMO is associated with low mood and negative affect. Therefore, poor mental health may be a cofounder of reaching for alcohol to self-soothing.

The obtained results allow for some comparisons between the general population struggling with FOMO and AUD patients. First, in both studied samples FOMO was a significant positive predictor of alcohol coping. Notably, this effect was more pronounced in the general sample. The discovery that FOMO played a significant role in both samples is a novel and noteworthy finding, aligning with the SDT framework. Up to this point, researchers had hypothesized that FOMO was linked to alcohol consumption primarily due to external social influences, which is a reasonable assumption in the context of young adults (Riordan et al., 2021). However, our observations regarding the role of FOMO in the sample of individuals with AUD, where age was not a significant factor, may suggest that internal, self-regulatory motives may also underlie the influence of FOMO on alcohol coping. Nevertheless, it remains a subject for future research to directly examine whether, in the case of individuals with AUD, FOMO is specific to social situations related to alcohol consumption, potentially termed as “alcohol-related FOMO” or ALFOMO (Al Abri, 2017). Additionally, it would be valuable to conduct a longitudinal study to investigate whether the level of FOMO decreases over the course of therapy, considering that individuals with AUD might fulfill their need for belonging by identifying with a group of people who struggle with the same kind of difficulties.

Our results have also considered the role of abstinence period in alcohol coping and health risk-taking in the clinical sample. It was evident that when it comes to alcohol coping, the abstinence period has the most pronounced negative impact. This suggests that the adoption and intensity of specific coping strategies change due to Cognitive Behavioral Therapy (CBT) and participation in the 12-step program (Magill et al., 2020), leading to increased utilization of adaptive coping mechanisms and reduced reliance on maladaptive ones (Cavicchioli et al., 2019). Interestingly, even after accounting for the crucial factor of abstinence duration in our model, the fear of missing out (FOMO) remained significant. Furthermore, in the context of health risk-taking, the abstinence period did not prove to be a significant factor. This observation may indicate that coping methods can improve during therapy, but the innate inclination toward risky behaviors is somewhat more resistant to change.

Despite the promising results, the current study is not without its limitations. Firstly, due to disparities in demographic characteristics between the general young adult population and clinical cohort, the potential for making direct statistical comparisons between these samples is restricted. Although we did compare regression coefficients between the groups, we did not include both samples in one analysis due to disparate samples characteristics. Secondly, the health risk-taking was measured only in the clinical sample, which additionally restricts the comparison potential between the two included samples. Thirdly, the pattern of alcohol usage in the general sample has not been controlled in the study. For this reason, there might be people in the general sample who do not drink at all as well as such who drink excessively. Future studies should account for this issue. Moreover, given the correlational design of the study, it has not been possible to establish causal relationships. Therefore, investigating the influence of FOMO on coping behaviors among AUD patients necessitates an experimental approach that delves into causality. Furthermore, to achieve a more profound comprehension of FOMO's role in alcohol-related coping strategies among clinical patients, conducting qualitative in-depth interviews is advisable. It is worth mentioning that the study was conducted on the Polish sample, which is ethnically homogenous, so it might be advisable to conduct it also in other, more ethnically diverse groups.

This study is of practical importance as it identifies the relationship between FOMO and adopting maladaptive coping strategies, specifically alcohol consumption. Given the prevalence of FOMO in younger generations (Jupowicz-Ginalska et al., 2021) it is crucial to acknowledge pronounced FOMO as its consequences might be severe, including alcohol coping and health risk-taking. Our findings are also pertinent for clinicians, they indicate that high FOMO in individuals with AUD could impede the use of constructive coping mechanisms. More broadly, our results suggest that the role of fear of missing out should not be overlooked in the prospective research involving clinical samples of AUD. It is worth considering the exploration of the area related to FOMO and the overuse of new technologies when collecting clinical interviews from individuals attending rehab centers.

**Compliance with ethical standards**

All procedures were conducted in accordance with the ethical standards of the institutional and/or Polish national research committees and with the 1964 Helsinki declaration and its later amendments or comparable ethical standards. Ethics Board of Faculty of Psychology, University of Warsaw approved the studies.

**Data availability statement**

Complete data for all studies can be found at the Open Science Framework: https://osf.io/xmpk3/?view_only=b02e6707aac84511baa3d4ce26c7127a

The original materials used to conduct this research are available upon e-mail request (diana.jaworska@psych.uw.edu.pl).

**Funding**

This research did not receive any specific grant from funding agencies in the public, commercial, or not-for-profit sectors.

## CRediT authorship contribution statement

**Diana Jaworska:** Conceptualization, Formal analysis, Investigation, Methodology, Project administration, Validation, Writing – original draft, Writing – review & editing. **Katarzyna Iwanicka:** Conceptualization, Investigation, Methodology, Validation, Writing – original draft, Writing – review & editing.

## Declaration of competing interest

The authors declare that they have no known competing financial interests or personal relationships that could have appeared to influence the work reported in this paper.

## Data Availability

The data is available here: https://osf.io/xmpk3/?view_only=b02e6707aac84511baa3d4ce26c7127a
