# Supplementary material for: Exploring the role of fear of missing out in coping and risk-taking among alcohol use disorder and general young adult populations
Source: Addict Behav Rep. 2024 Jan 23;19:100532. doi: 10.1016/j.abrep.2024.100532 (PMC10835594; doi:10.1016/j.abrep.2024.100532)
Supplement: Supplementary Data 1 [file mmc1.docx]

**Appendix 1. Supplementary material.**

**Exploring the role of fear of missing out in coping and risk-taking among alcohol use disorder and general young adult populations.**

1. **Materials used in Study 1 and 2^[[1]](#footnote-1)^.**

- **FOMO Scale (Przybylski et al., 2013)**

Below is a collection of statements about your everyday experience. Using the scale provided please indicate how true each statement is of your general experiences. Please answer according to what really reflects your experiences rather than what you think your experiences should be. Please treat each item separately from every other item.

*Not at all true of me | 1*

*Slightly true of me | 2*

*Moderately true of me | 3*

*Very true of me | 4*

*Extremely true of me | 5*

1. I fear others have more rewarding experiences than me.

2. I fear my friends have more rewarding experiences than me.

3. I get worried when I find out my friends are having fun without me.

4. I get anxious when I don't know what my friends are up to.

5. It is important that I understand my friends "in jokes."

6. Sometimes, I wonder if I spend too much time keeping up with what is going on.

7. It bothers me when I miss an opportunity to meet up with friends.

8. When I have a good time it is important for me to share the details online (e.g. updating

status).

9. When I miss out on a planned get-together it bothers me.

10. When I go on vacation, I continue to keep tabs on what my friends are doing.

- **Mini-COPE Scale (Juczyński, Ogińska-Bulik, 2009)**

People react differently when they encounter difficult or stressful events in their lives. You can deal with stress advise in a variety of ways. The questionnaire is designed to establish how you usually behave when you experience these events. It is understandable that different events trigger different reactions, but what do you usually do when you are experiencing a lot unpleasant event? In each line, mark one answer that suits you best, circling it around the appropriate digit (0-1-2-3). Do not omit any statement.

The individual numbers mean:

*0 = “I hardly ever do this”,*

*1 = “I rarely do this”,*

*2 = “I do this often”,*

*3 = “I almost always do this”.*

**When I am in a very difficult situation, it is usually:**

1. I do work or other activities so as not to think about it. 0 1 2 3

2. My efforts are focused on doing something about the situation. 0 1 2 3

3. I say to myself "that's not true". 0 1 2 3

4. I drink alcohol or take other measures to make me feel better. 0 1 2 3

5. I get emotional support from others. 0 1 2 3

6. I give up trying to achieve the goal. 0 1 2 3

7. I am taking action to improve the situation. 0 1 2 3

8. I don't want to believe this really happened. 0 1 2 3

9. I'm talking about things that help me get away from unpleasant feelings. 0 1 2 3

10. I am looking for advice and help from others on what to do. 0 1 2 3

11. I drink alcohol or take other measures to help me get through this. 0 1 2 3

12. I am trying to see it in a different, more positive light. 0 1 2 3

13. I criticize myself. 0 1 2 3

14. I am trying to develop a strategy or plan defining what to do. 0 1 2 3

15. I receive encouragement and understanding from others. 0 1 2 3

16. I give up on dealing with it. 0 1 2 3

17. I am looking for good points in what happened. 0 1 2 3

18. I'm just kidding about this. 0 1 2 3

19. I do something to think less about it, e.g. go to the cinema, watch TV, read, I am daydreaming, sleeping or shopping. 0 1 2 3

20. I accept that this has already happened. 0 1 2 3

21. I reveal my negative emotions. 0 1 2 3

22. I am trying to find solace in religion or in my faith. 0 1 2 3

23. I get help or advice from other people. 0 1 2 3

24. I'm learning to live with it. 0 1 2 3

25. I am seriously wondering what steps should be taken. 0 1 2 3

26. I blame myself for what happened. 0 1 2 3

27. I pray or meditate. 0 1 2 3

28. I treat this situation as playing. 0 1 2 3

- **Health risk-taking (subscale of DOSPERT Scale, Blais, Weber, 2006)^[[2]](#footnote-2)^**

For each of the following statements, please indicate your likelihood of engaging in each activity or behavior. Provide a rating from 1 to 5, using the following scale:

_________________________________­­­_______________________

1 2 3 4 5

Very Unlikely Not sure Likely Very

unlikely likely

1. Consuming excessive amount of alcohol in a social situation. (H) _____
2. Engaging in unprotected sex. (H) _____
3. Not wearing a seatbelt when being a passenger in the front seat. (H) _____
4. Not wearing a helmet when riding a motorcycle. (H) _____
5. Exposing yourself to the sun without using sunscreen. (H) _____
6. Walking home alone at night in a somewhat unsafe area of town. (H) _____

- **Sociodemographic questions**

1. Year of birth: (open question)

2. Gender: (Female, Male, Other)

3. Education: primary, secondary, higher

4. Place of residence: village, city up to 50,000, city from 50,000 to 150,000, city from 150,000 to 500,000, city with more than 500 thousand

- **Alcohol addiction related questions**^2^

5. Age of alcohol initiation [in years]:

6. Duration of abstinence [in months]:

7. Were any of your parents addicted to alcohol?

- Yes

- No

8. Have you been ordered to undergo drug addiction treatment by a court?

- Yes

- No

9. How many times have you been in alcohol therapy? [open question]:

**2) Descriptive statistics.**

**Table 1. Descriptive statistics for the general sample (Study 1).**

| **Variable** | **N** | **M** | **SD** | **Min.** | **Max.** | **Skewness** |
| --- | --- | --- | --- | --- | --- | --- |
| FOMO | 356 | 2.71 | .78 | 1.00 | 5.00 | -.15 |
| Alcohol coping | 356 | .76 | .85 | 0.00 | 3.00 | .74 |
| Self-blame | 356 | 1.53 | .83 | 0.00 | 3.00 | .05 |
| Behavioral disengagement | 356 | 1.14 | .71 | 0.00 | 3.00 | .12 |
| Self-distraction | 356 | 1.65 | .65 | 0.00 | 3.00 | -.34 |
| Denial | 356 | 1.15 | .78 | 0.00 | 3.00 | .21 |
| Emotional support | 356 | 1.49 | .78 | 0.00 | 3.00 | -.19 |
| Instrumental support | 356 | 1.51 | .79 | 0.00 | 3.00 | -.13 |
| Active coping | 356 | 1.82 | .65 | 0.00 | 3.00 | -.24 |
| Planning | 356 | 1.75 | .68 | 0.00 | 3.00 | -.23 |
| Acceptance | 356 | 1.76 | .62 | 0.00 | 3.00 | -.48 |
| Positive reframing | 356 | 1.56 | .69 | 0.00 | 3.00 | -.44 |
| Religion | 356 | .92 | .88 | 0.00 | 3.00 | .54 |
| Venting | 356 | 1.48 | .64 | 0.00 | 3.00 | -.13 |
| Humor | 356 | 1.12 | .72 | 0.00 | 3.00 | .24 |

**Table 2. Descriptive statistics for the clinical sample (Study 2).**

| **Variable** | **N** | **M** | **SD** | **Min.** | **Max.** | **Skewness** |
| --- | --- | --- | --- | --- | --- | --- |
| FOMO | 66 | 2.27 | .73 | 1.00 | 4.50 | .79 |
| Alcohol coping | 66 | 1.21 | 1.16 | .00 | 3.00 | .32 |
| Self-blame | 66 | 1.42 | .69 | .00 | 3.00 | .06 |
| Behavioral disengagement | 66 | 1.06 | .71 | .00 | 3.00 | .38 |
| Self-distraction | 66 | 1.70 | .66 | .00 | 3.00 | -.18 |
| Denial | 66 | .82 | .74 | .00 | 3.00 | .66 |
| Emotional support | 66 | 1.58 | .93 | .00 | 3.00 | -.12 |
| Instrumental support | 66 | 1.60 | .89 | .00 | 3.00 | .07 |
| Active coping | 66 | 1.94 | .59 | .50 | 3.00 | -.05 |
| Planning | 66 | 1.94 | .59 | 1.00 | 3.00 | .12 |
| Acceptance | 66 | 1.89 | .56 | 1.00 | 3.00 | .22 |
| Positive reframing | 66 | 1.62 | .69 | .00 | 3.00 | -.06 |
| Religion | 66 | .80 | .96 | .00 | 3.00 | .93 |
| Venting | 66 | 1.58 | .53 | .00 | 2.50 | -.42 |
| Humor | 66 | 1.16 | .73 | .00 | 3.00 | .09 |
| Health risk-taking | 66 | 3.44 | 1.41 | 1.00 | 6.83 | .31 |

1. **Additional statistical analyses – moderation effect of age.**

In order to test for the boundary conditions of the link between FOMO and alcohol coping, the moderation effect of age was tested with the use of Model 1 of Hayes’s PROCESS macro (Hayes, 2018). First, the model for the general sample (Study 1) was created. The interaction effect was not significant (*p* = .07; Table 3). Next, the analogous model for the clinical sample was created. The interaction effect was insignificant as well (*p* = .31; Table 3). It indicates that the relationship between FOMO and alcohol coping is not moderated by the age of a participant.

**Table 3.** Moderation analyses.

|  | ***Coeff.*** | ***SE*** | ***t*** | ***R*** | | ***R^2^*** | | | ***F*** | | ***df1*** | | ***df2*** | | |  |  |  |
| --- | --- | --- | --- | --- | --- | --- | --- | --- | --- | --- | --- | --- | --- | --- | --- | --- | --- | --- |
| *Study 1 – general population* | | | | .44 | | .19 | | | 28.171*** | | 3 | | 352 | | |  |  |  |
| Constant | .661 | .817 | .809 |  | | |  | |  | |  | | | |  | | |  |
| FOMO | -.051 | .293 | -.175 | |  | |  | | |  | |  | |  | | |  |  |
| Age | -.042 | .029 | -1.462 | |  | |  | | |  | |  | |  | | |  |  |
| FOMO x Age | .019 | .010 | .071 | |  | |  | | |  | |  | |  | | |  |  |
| *Study 2 – clinical sample* | | | | .45 | | .20 | | | 5.676** | | 3 | | 68 | | |  |  |  |
| Constant | 2.495 | 1.683 | 1.482 |  | | |  |  | |  |  |  |  |  |  |  |  |  |
| FOMO | -.006 | .674 | -.193 |  | | |  |  | |  |  |  |  |  |  |  |  |  |
| Age | .001 | .041 | -1.506 |  | | |  |  | |  |  |  |  |  |  |  |  |  |
| FOMO x Age | .439 | .017 | 1.016 |  | | |  |  | |  |  |  |  |  |  |  |  |  |

1. In our studies, the Polish translation of all scales was used. [↑](#footnote-ref-1)
2. Used only in Study 2. [↑](#footnote-ref-2)
